# ChaC1-based drug screenings identify a synergistic lethal effect of auranofin and proteasome inhibitors in hepatocellular carcinoma cells

**DOI:** 10.1038/s41420-025-02838-6

**Published:** 2025-11-17

**Authors:** Cheng Yu, Jinyu Liu, Hejia Jian, Biwen Wen, Mingting Jiang, Binghui Zhang, Meiyan Lin, Junjin Lin, Zichan Dai, Chuan-Qi Zhong, Min Zheng

**Affiliations:** 1 Fujian Key Laboratory of Cognitive Function and Diseases, The School of Basic Medical Sciences, Fujian Medical University, Fujian, China; 2 Public Technology Service Center, Fujian Medical University, Fujian, China; 3 Department of Biochemistry and Molecular Biology, The School of Basic Medical Sciences, Fujian Medical University, Fujian, China; 4 State Key Laboratory of Cellular Stress Biology, Innovation Center for Cell Signaling Network, School of Life Sciences, Xiamen University, Xiamen, Fujian China

**Keywords:** Cell death, Cancer therapy

## Abstract

The γ-glutamylcyclotransferase, ChaC1, is an enzyme catalyzing glutathione (GSH) degradation. In this study, we performed a ChaC1-activity-directed pharmacological screening from FDA-approved drug library to identify GSH-sensitive agents, and found that ChaC1 overexpression mediated glutathione depletion largely enhanced auranofin (AUR)-induced cell death in hepatocellular carcinoma (HCC) cells. AUR elicited sustained activation of oxidative and endoplasmic reticulum (ER) stress pathways in ChaC1-overexpressed HCC cells, exemplified by Nrf2 and ATF4 upregulation. Proteomic analyses identified DNA Damage Inducible Transcript 4 (DDIT4) as one of the key pro-death effectors in this process. Mechanistic investigation revealed ChaC1/AUR-driven cytotoxicity was attenuated by disulfide reducing agents (e.g., NAC and TCEP), while maintaining resistance to canonical programmed death pathway inhibitors targeting apoptosis, necroptosis, and ferroptosis. To mimic ChaC1 overexpression by inducing endogenous ChaC1 high expression, a complementary ChaC1-induction-based screening was performed and revealed proteasome inhibitors (e.g., bortezomib, ixazomib, delanzomib) as potent inducers of endogenous ChaC1 via ATF4-dependent transcriptional activation. Combinational treatment of AUR and proteasome inhibitor (PI) synergistically led to catastrophic cell death, reversible by NAC or protein synthesis inhibitor CHX, yet refractory to blockade of apoptosis, necroptosis or ferroptosis. And PI/AUR co-treatment recapitulated the phenotype of DDIT4 induction, while genetic disruption of the ATF4-ChaC1 axis abolished both cell death and DDIT4 upregulation. This functional convergence demonstrates that ChaC1 activation, either achieved through genetic manipulation or pharmacological induction, creates a synthetic lethal context for AUR in HCC cells. Together, our study establishes a ChaC1-based pharmacological discovery platform that identifies proteasome inhibitor and auranofin combination as a mechanistically rational anti-HCC strategy, providing both methodological innovation in drug repurposing screening and immediate translational potential for HCC treatment.

## Introduction

Hepatocellular carcinoma (HCC), representing approximately 90% of primary liver malignancies, has become the second most prevalent cause of cancer-associated mortality worldwide [1, 2]. Despite its clinical significance, therapeutic options for advanced HCC remain substantially limited [3]. The liver’s unique metabolic profile warrants particular attention, being the principal site of glutathione (GSH) biosynthesis. This tripeptide plays critical cytoprotective functions through dual mechanisms of xenobiotic detoxification and redox homeostasis maintenance, effectively shielding hepatocytes from multiple regulated cell death pathways including ferroptosis [4, 5], cuproptosis [6] and disulfidptosis [7, 8]. Emerging preclinical evidence suggests that targeted depletion of this endogenous antioxidant system may potentiate therapeutic efficacy in hepatic malignancies, implicating glutathione depletion as a good strategy to enhance HCC therapy [9, 10].

The glutathione specific γ-glutamylcyclotransferase, ChaC1, catalyzes the proteolytic cleavage of glutathione into 5-oxoproline and the cysteine-glycine dipeptide, thereby driving intracellular glutathione depletion [11]. ChaC1 is highly conserved in human and mouse, exhibiting 88% amino acid sequence homology. Structural-functional analyses reveal that the conserved catalytic glutamate residue (human E115/murine E116) is essential for enzymatic activity, as its substitution with glutamine completely abrogates glutathione degradation capacity [11]. Mechanistically, ChaC1 expression is transcriptionally regulated through the ATF4-mediated unfolded protein response pathway during endoplasmic reticulum (ER) stress [12, 13]. Importantly, elevated ChaC1 levels have been pathophysiologically associated with ER stress-induced cytotoxicity across multiple experimental models, suggesting its functional involvement in stress-mediated cell death execution [5, 14].

To employ ChaC1’s glutathione-depleting activity, we designed a two-phase ChaC1-based drug screening assay for anti-HCC agents from the United States Food and Drug Administration (FDA)-approved drug library. Primary screening found that ChaC1 overexpression mediated glutathione depletion dramatically enhanced the anti-cancer effect of auranofin (AUR), a gold (I)-containing compound approved by FDA since 1980’s to treat rheumatoid arthritis (RA) [15]. To recapitulate this therapeutic vulnerability through endogenous pathway activation, we performed a secondary screening for ChaC1-inducing agents, revealing proteasome inhibitors, such as bortezomib (BTZ), ixazomib (IXZ), and delanzomib (DLZ), as potent inducers of endogenous ChaC1. These proteasome inhibitors are being used for the treatment of multiple myeloma and mantle cell lymphoma [16]. As expected, proteasome inhibitors enhanced AUR cytotoxicity through inducing ChaC1 expression. Together, through ChaC1-based drug screening strategy, we identified a synergistic lethal effect of auranofin and proteasome inhibitors in HCC cells, suggesting that combination of these drugs might be repurposed for HCC treatment.

## Results

### ChaC1-activity-based drug screening identifies auranofin as a potent cell death inducer in glutathione-depleted HCC cells

**Fig. 1:**
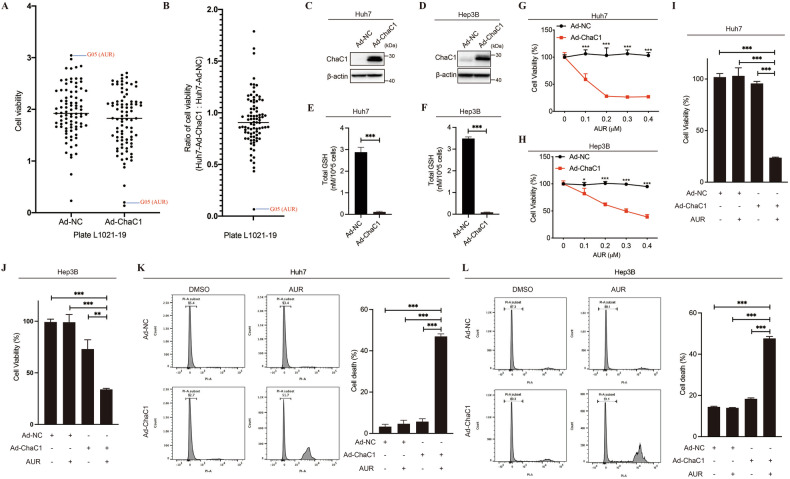
ChaC1 overexpression sensitizes AUR-induced cell death in HCC cells. **A**, **B** Huh7 cells were infected with Ad-NC or Ad-ChaC1 (1×10^8^ pfu/mL). 24 h later, cells were treated with 1 µM drugs from FDA-approved drug library (Plate L1021-19). 24 h later, cell viabilities were measured by CCK8 assay (**A**). The ratios of cell viabilities of Ad-ChaC1 infected cells to Ad-NC infected cells were calculated and plotted in (**B**). (C ~ F) Huh7 and Hep3B cells were infected with Ad-NC or Ad-ChaC1 (1×10^8^ pfu/mL) for 24 h. The expression levels of ChaC1 were determined by western blotting (**C, D**). Intracellular glutathione levels in Huh7 and Hep3B were measured by Tietze’s recycling assay (**E**, **F**). **G**, **H** Huh7 (**G**) and Hep3B (**H**) cells were infected with 1×10^8^ pfu/mL Ad-NC or Ad-ChaC1 for 24 h, and then treated with increasing doses of AUR for 24 h. Cell viabilities were measured by CCK8 assay. **I**–**L** Huh7 (**I**, **K**) and Hep3B (**J**, **L**) cells were infected with adenovirus for 24 h and then treated with 0.3 µM AUR for 24 h. Cell viability was measured by CCK8 assay (**I, J)**. Cell death was measured by propidium iodide staining (1 µg/mL) followed by flow cytometric analysis (**K**, **L**).

While GSH mediates drug detoxification, therapeutic vulnerabilities affected by GSH remain poorly defined in HCC. To systematically identify GSH-sensitive chemotherapeutics, we constructed GSH-depleted HCC cells through infection of ChaC1-expressing adenovirus (Ad-ChaC1). High-throughput screening from FDA-approved drug library using cell viability assay showed that auranofin (AUR) had a much more striking anti-cancer efficacy in ChaC1-overexpressed Huh7 cells than that in control cells (Fig. 1A, B), suggesting that AUR is one of the GSH-sensitive drugs. As expected, ChaC1 overexpression almost depleted glutathione in Huh7 and Hep3B cells (Fig. 1C–F). Further investigation validated that ChaC1 overexpression greatly sensitized Huh7 and Hep3B cells to AUR cytotoxicity in a dose dependent manner (Fig. 1G–J). Compared to the control cells, the IC50 of AUR was reduced almost 8 – 10 fold in Huh7 cells infected with Ad-ChaC1 (Supplementary Fig. 1). Cell death assay also showed that AUR at submicromolar concentration (<1 μM) was sufficient to cause cell death in ChaC1-overexpressed Huh7 and Hep3B cells, but not in the control cells (Fig. 1K, L). These findings implicate a functional codependency between GSH depletion and AUR sensitivity, redefining ChaC1 as a therapeutic target for precision redox interventions in HCC.

### The effect of ChaC1 overexpression on AUR-induced cell death requires its gamma-glutamylcyclotransferase activity

**Fig. 2:**
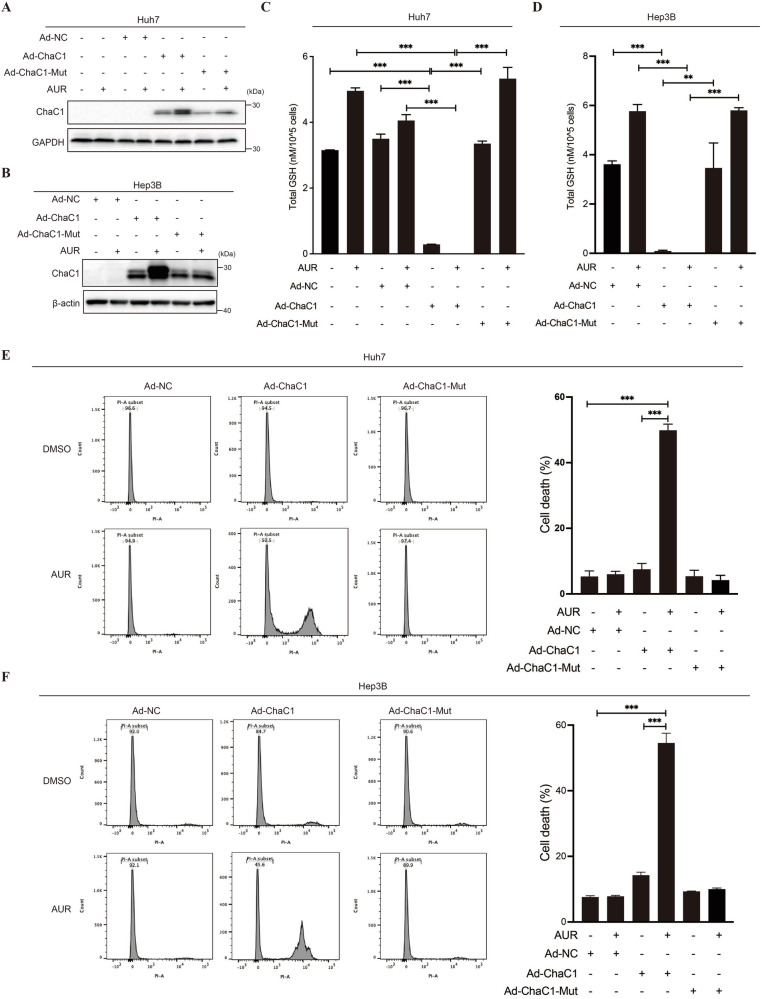
The effect of ChaC1 overexpression on AUR-induced cell death requires its gamma-glutamylcyclotransferase activity. **A, B** Huh7 (**A**) and Hep3B (**B**) cells were infected with 1×10^8^ pfu/mL Ad-NC, Ad-ChaC1 or Ad-ChaC1-Mut for 24 h, and then treated with 0.3 µM AUR for 12 h. The expression levels of ChaC1 were determined by western blotting. **C**, **D** Intracellular glutathione levels were measured by Tietze’s recycling assay in Huh7 (**C)** and Hep3B (**D**) cells. **E**, **F** Cell death was measured by propidium iodide staining (1 µg/mL) followed by flow cytometric analysis in Huh7 (**E**) and Hep3B (**F**) cells.

To investigate whether ChaC1 enzyme activity was essential for its effect on AUR-induced cell death, we constructed a ChaC1 enzyme-dead mutant expressing adenovirus (Ad-ChaC1-Mut) through site-directed mutagenesis of ChaC1’s catalytic glutamate residue. Adenoviral delivery of this catalytically null mutant (Ad-ChaC1-Mut) in Huh7/Hep3B systems (Fig. 2A, B) preserved basal glutathione levels (Fig. 2C, D), in contrast to wild-type ChaC1. Notably, low-dose of AUR (0.3 μM) elicited glutathione elevation in ChaC1-Mut-expressing cells, which is consistent with the previous data [17]. And this catalytic inactivation completely abrogated its effect on AUR cytotoxicity in HCC cells, as evidenced by cell death assays (Fig. 2E, F). The absolute requirement for γ-glutamylcyclotransferase activity implicates that ChaC1-mediated glutathione catabolism, rather than its protein-protein interactions, drives therapeutic sensitization. These findings demonstrate that the glutathione degradation enzyme activity is essential for ChaC1 to sensitize HCC cells to AUR cytotoxicity.

### AUR induces acute oxidative stress and ER stress in ChaC1-overexpressed cells

**Fig. 3:**
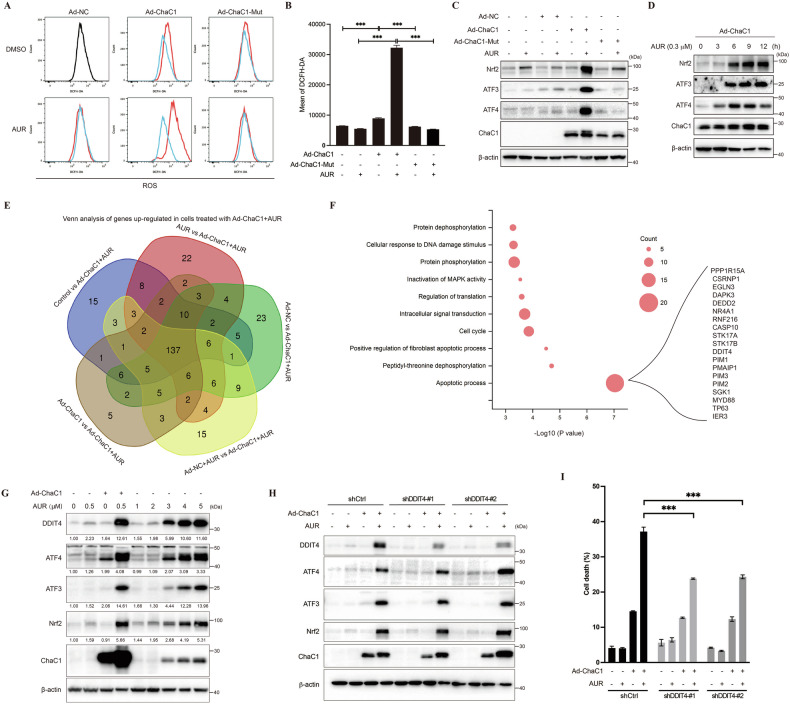
Ad-ChaC1 and AUR co-treatment leads to acute cellular stress responses. **A, B** Huh7 cells were infected with 1×10^8^ pfu/mL Ad-NC, Ad-ChaC1 or Ad-ChaC1-Mut for 24 h. Cells were then treated with 0.3 µM AUR for 12 h. Reactive oxygen species levels were measured by DCFH-DA staining followed by flow cytometric analysis (**A**). Quantitative analysis was performed (**B**). **C** The expression levels of Nrf2, ATF3, ATF4 and ChaC1 were determined by western blotting. **D** Huh7 cells were infected with 1×10^8^ pfu/mL Ad-ChaC1 for 24 h and then treated with 0.3 µM AUR for increasing time. The expression levels of Nrf2, ATF3, ATF4 and ChaC1 were determined by western blotting. **E** Proteomic profiling was conducted across six experimental conditions, including untreated controls, AUR monotherapy, Ad-NC infection alone, Ad-ChaC1 infection alone, Ad-NC + AUR co-treatment and Ad-ChaC1 + AUR co-treatment. Venn diagram analysis of differentially expressed proteins revealed 137 upregulated targets shared across Ad-ChaC1 and AUR co-treated groups. **F** The genes of interest (137 up-regulated genes) were input into DAVID Bioinformatics Resources (http://david.ncifcrf.gov). Analysis of Gene Ontology was shown. **G** Cells were treated as indicated. The protein expression levels of DDIT4, ATF4, ATF3, Nrf2 and ChaC1 were determined by western blotting. **H** Huh7 cells were infected with lentivirus expressing control shRNA or DDIT4-shRNA. The expression levels of DDIT4, ATF4, ATF3, Nrf2 and ChaC1 were determined by western blotting. **I** DDIT4-knockdown Huh7 cells and the control cells were infected with 1×10^8^ pfu/mL Ad-NC or Ad-ChaC1 for 12 h, and then treated with 0.3 µM AUR for 24 h. Cells were stained with 1 µg/mL propidium iodide followed by flow cytometric analysis.

To further elucidate the underlying mechanism, we hypothesized that dual targeting of glutathione (ChaC1) and thioredoxin (AUR) systems would lead to catastrophic redox collapse. Flow cytometric quantification of DCFH-DA fluorescence confirmed acute ROS increased exclusively in ChaC1/AUR co-treated HCC cells, but absent in catalytically inactive ChaC1-Mut/AUR co-treated and control cells (Fig. 3A, B). Western blotting results revealed coordinated upregulation of stress-responsive transcription factors, such as redox sensor Nrf2, ER stress effector ATF4, and integrated stress response mediator ATF3 (Fig. 3C). Time course analysis showed that upregulation of Nrf2, ATF3 or ATF4 occurred at 6 h post treatment (Fig. 3D). Because ATF4, ATF3 and Nrf2 are all transcriptional factors, these data indicate that a series of genes might be induced by AUR treatment in ChaC1-overexpressed cells. Proteomic analysis identified 137 genes uniquely upregulated in ChaC1/AUR co-treated cells (Fig. 3E). Gene ontology (GO) analysis showed that among these genes, 19 genes were involved in cell death process, including DNA Damage Inducible Transcript 4 (DDIT4) [18] (Fig. 3F). Strikingly, ChaC1 overexpression induced 8–10-fold pharmacodynamic amplification, enabling AUR at submicromolar concentration to achieve DDIT4 induction equivalent to AUR monotherapy at 5 μM (Fig. 3G). Induction of DDIT4 by co-treatment of AUR and Ad-ChaC1 was also observed in Hep3B and PLC/PRF/5 cells, implicating that this mechanism is consistent across HCC cell lines (Supplementary Fig. 2). Furthermore, knockdown of DDIT4 suppressed AUR-induced cell death in ChaC1-overexpressed Huh7 cells (Fig. 3H, I), suggesting DDIT4 was a key pro-death effector of ChaC1/AUR-induced cytotoxicity in HCC cells.

### AUR-induced cell death in ChaC1-overexpressed cells is attenuated by disulfide reducing agents

**Fig. 4:**
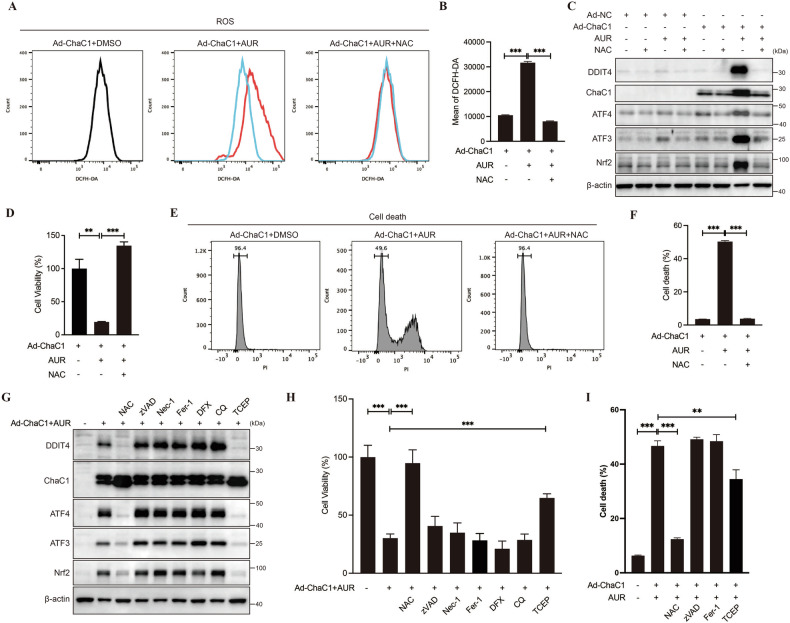
Disulfide reducing agents attenuate AUR-induced Cell death in ChaC1-overexpressed cells. **A, B** Huh7 cells were infected with 1×10^8^ pfu/mL Ad-ChaC1 for 24 h. Cells were pre-treated with 5 mM NAC for 1 h. And then cells were treated with 0.3 µM AUR for 12 h. ROS levels were measured by DCFH-DA staining followed by flow cytometric analysis. **C** The expression levels of DDIT4, ChaC1, ATF4, ATF3 and Nrf2 were determined by western blotting. **D** Cell viabilities were measured by CCK8 assay. **E, F** Cell death assays were performed. **G** Huh7 cells were infected with 1×10^8^ pfu/mL Ad-ChaC1 for 24 h. Cells were pre-treated with cell death inhibitors (zVAD: 10 µM, Nec-1: 10 µM, Fer-1: 10 µM, DFX: 200 µM, CQ: 10 µM, TCEP: 5 mM) for 1 h and then treated with 0.5 µM AUR for 12 h. The expression levels of DDIT4, ChaC1, ATF4, ATF3 and Nrf2 were determined by western blotting. **H** Cell viabilities were measured by CCK8 assays. **I** Cell death was measured by propidium iodide staining (1 µg/mL) followed by flow cytometric analysis.

N-Acetyl-L-cysteine (NAC) is a disulfide reducing antioxidant derived from the amino acid L-cysteine, which is required to produce glutathione. Flow cytometric analysis showed that NAC suppressed AUR-induced ROS accumulation in ChaC1-overexpressed cells (Fig. 4A, B). And AUR-induced expression of DDIT4, ATF4, ATF3 and Nrf2 were largely attenuated by NAC pretreatment (Fig. 4C). Furthermore, NAC pretreatment fully rescued ChaC1/AUR-driven cytotoxicity, as indicated by cell viability assay (Fig. 4D) and cell death assay (Fig. 4E, F). Notably, this cytotoxicity process demonstrated absolute independence from canonical death pathways, as pharmacological blockade of apoptosis (zVAD), necroptosis (Nec-1), ferroptosis (Fer-1/DFX), or autophagy (CQ) failed to attenuate cellular stress and cell death (Fig. 4G, H). Furthermore, this cytotoxic process exhibited redox dependency distinct from classical ROS-mediated pathways, as evidenced by the ineffectiveness of Trolox (cytosolic ROS scavenger) and MitoTEMPO (mitochondrial ROS inhibitor) (Supplementary Fig. 3). Interestingly, the thiol-independent disulfide reductant Tris (2-carboxyethyl) phosphine (TCEP) recapitulated NAC’s cytoprotective effects (Fig. 4G–I). This dual pharmacological evidence suggests that disulfide stress, but not general or mitochondria ROS, underlies AUR cytotoxicity in ChaC1-overexpressed cells. We also examined GSH/GSSG ratio that more accurately reflects cellular redox balance and disulfide stress. As shown in Supplementary Fig. 4, co-treatment of AUR with Ad-ChaC1 dramatically decreased the ratio of GSH/GSSG in HCC cells. These collective data delineate a previously unrecognized regulated cell death modality in HCC cells, uniquely contingent on disulfide dysregulation and reversible by disulfide reduction.

### ChaC1-induction-based drug screening identifies proteasome inhibitors as ChaC1-inducing drugs in HCC cells

**Fig. 5:**
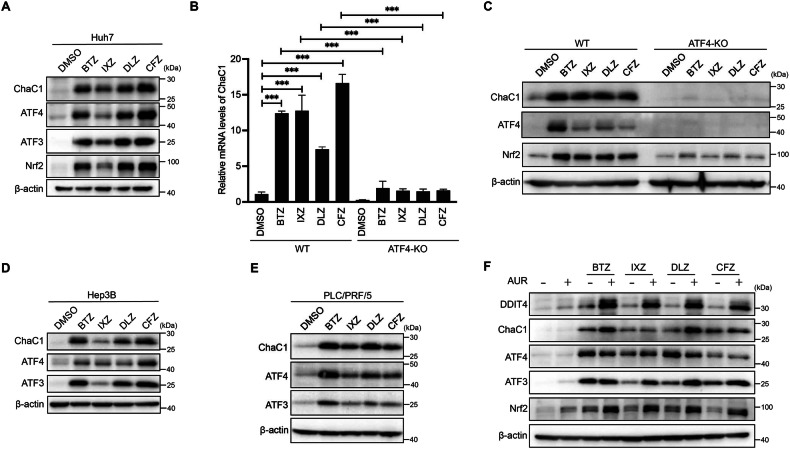
Proteasome inhibitors induce endogenous ChaC1 and enhance AUR-induced cell death in HCC cells. **A** Huh7 cells were treated with 50 nM BTZ, 100 nM IXZ, 100 nM DLZ or 100 nM CFZ for 12 h. The protein expression levels of ChaC1, ATF4, ATF3 and Nrf2 were determined by western blotting. **B**, **C** ATF4 knockout Huh7 cells and their control cells were treated with BTZ, IXZ, DLZ or CFZ for 12 h. The mRNA levels of ChaC1 were measured by qRT-PCR (**B**). The protein expression levels of ChaC1, ATF4 and Nrf2 were determined by western blotting (**C**). **D**, **E** Hep3B (**D**) and PLC/PRF/5 (**E**) cells were treated with BTZ, IXZ, DLZ or CFZ for 12 h. The expression levels of ChaC1, ATF4 and ATF3 were measured by western blotting. **F** Huh7 cells were treated with 1 µM AUR with or without BTZ, IXZ, DLZ or CFZ for 12 h. The expression levels of DDIT4, ChaC1, ATF4, ATF3 and Nrf2 were determined by western blotting.

The data above demonstrated that overexpression of ChaC1 would sensitize HCC cells to AUR-induced cell death. Thus, we proposed that induction of endogenous ChaC1 might also enhance AUR-induced cell death in HCC cells. A drug screening was then performed for ChaC1-inducing drugs from the FDA-approved drug library. Interestingly, a family of proteasome inhibitors including bortezomib (BTZ), ixazomib (IXZ, MLN9708/MLN2238) and delanzomib (DLZ, CEP-18770) potently induced endogenous ChaC1 expression in Huh7 cells (Supplementary Fig. 5). Further western blotting and quantitative real-time PCR analysis demonstrated that these proteasome inhibitors, as well as carfilzomib (CFZ), induced both protein and mRNA levels of ChaC1 in Huh7 cells (Fig. 5A, B). Mechanistically, PI-driven ChaC1 induction largely depended on ATF4, as CRISPR/Cas9-mediated ATF4 knockout abrogated this response (Fig. 5B, C). Induction of ChaC1 by proteasome inhibitors was also observed in Hep3B and PLC/PRF/5 cells (Fig. 5D, E). Mirroring AUR treatment in ChaC1-overexpressed cells, induction of DDIT4 and Nrf2 was also observed in Huh7 cells co-treated with proteasome inhibitors and AUR (Fig. 5F).

### Proteasome inhibitors and AUR synergistically induce cell death in HCC cells

**Fig. 6:**
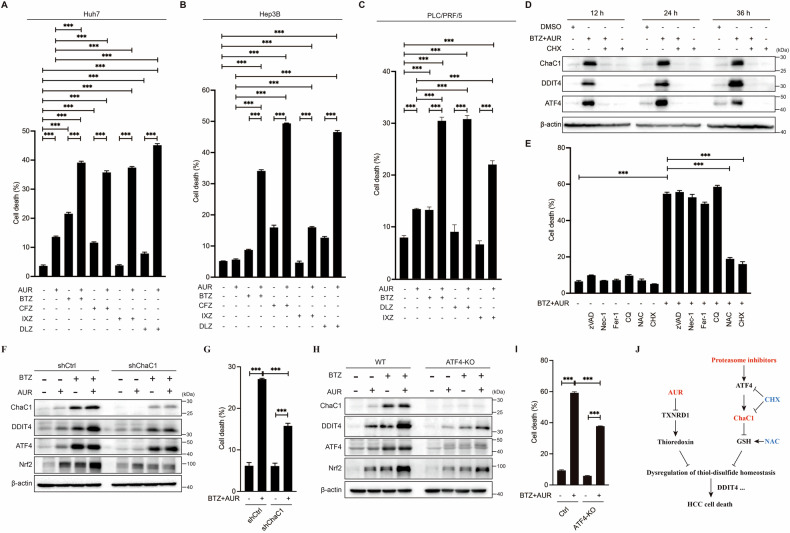
BTZ enhances the anti-HCC efficacy of AUR through induction of ChaC1. **A–C** Huh7 (**A**), Hep3B (**B**) and PLC/PRF/5 (**C**) cells were treated as indicated for 36 h. Cells were stained with 1 µg/mL propidium iodide followed by flow cytometric analysis. **D** Huh7 cells were treated with 50 nM BTZ and 1 µM AUR for 12, 24 or 36 h, with/without 5 µM CHX pretreatment for 1 h. The protein expression levels of ChaC1, DDIT4 and ATF4 were determined by western blotting. **E** Huh7 cells were pretreated with or without cell death inhibitors (10 µM zVAD, 10 µM Nec-1, 10 µM Fer-1, 10 µM CQ, 5 mM NAC or 5 µM CHX) for 1 h, and then treated with 50 nM BTZ and 1 µM AUR for 36 h. Cells were stained with 1 µg/mL propidium iodide followed by flow cytometric analysis. **F**, **G** ChaC1 knock-down (KD) and its control Huh7 cells were treated with 50 nM BTZ and 1 µM AUR for 36 h. The protein expression levels of ChaC1, DDIT4, ATF4 and Nrf2 were determined by western blotting (**F**). Cell death assay was performed (**G**). **H**, **I** ATF4-KO Huh7 cells and their control Huh7 cells were treated with 50 nM BTZ and 1 µM AUR for 36 h. The protein expression levels of ChaC1, DDIT4, ATF4 and Nrf2 were determined by western blotting (**H**). Cell death was measured by propidium iodide (1 µg/mL) staining and flow cytometric analysis (**I**). **J** Graphical Abstract: Overexpression of exogenous ChaC1 or induction of endogenous ChaC1 by proteasome inhibitors enhances AUR-induced HCC cell death by synergistically triggering dysregulation of thiol-disulfide homeostasis.

Next, we assessed their combined effects on cell death. As indicated by cell death assay, combinational treatment of PI with AUR markedly enhanced HCC cell death compared to monotherapy (Fig. 6A–C), recapitulating the sensitization phenotype observed in AUR/ChaC1 models. These data suggest that PI-mediated induction of endogenous ChaC1 functionally mimics genetic overexpression of ChaC1 to potentiate AUR cytotoxicity. To evaluate potential therapeutic synergism, we measured the combination index of BTZ and AUR using the online SynergyFinder software (https://synergyfinder.fimm.fi). As shown in Supplementary Fig. 6, the highest zero interaction potency (ZIP) synergy score for BTZ and AUR is 13.978, implicating that BTZ and AUR have strong synergistic anti-HCC effect.

To investigate whether induction of ChaC1 was essential for the proteasome inhibitors to enhance AUR-induced cell death, we used cycloheximide (CHX) to suppress de novo protein synthesis. CHX pretreatment abolished both ATF4/ChaC1/DDIT4 upregulation and cell death induced by co-treatment of AUR and proteasome inhibitor (e.g., bortezomib) (Fig. 6D, E). Notably, this cytotoxicity process remained resistant to inhibitors of apoptosis (zVAD), necroptosis (Nec-1), ferroptosis (Fer-1), and autophagy (CQ), further distinguishing it from established death modes. By contrast, NAC suppressed the cell death induced by co-treatment of AUR and BTZ (Fig. 6E). And GSH/GSSG analysis showed that co-treatment of AUR and BTZ also led to dramatic decrease of GSH/GSSG (Supplementary Fig. 7). These data again implicated that the cytotoxicity might be due to disulfide-mediated cell death. To investigate the role of ATF4-ChaC1 pathway in the cell death induced by co-treatment of AUR and BTZ, we constructed ATF4 or ChaC1-deficient Huh7 cell lines. Cell death assay showed that ATF4 or ChaC1 depletion largely inhibited DDIT4 upregulation and cell death induced by BTZ and AUR co-treatment (Fig. 6F–I). These results delineate a sequential mechanism wherein PI-driven ATF4 activation induces ChaC1 expression, by which proteasome inhibitor and AUR synergistically induce HCC cell death.

## Discussion

Targeting glutathione (GSH) depletion has emerged as a therapeutically exploitable strategy to potentiate chemotherapeutic responses. Our study shows that central to this approach could be ChaC1, a cytoplasmic γ-glutamylcyclotransferase executing rate-limiting proteolytic cleavage of GSH. In this study, we systematically demonstrated that both genetic amplification (exogenous overexpression) and pharmacological induction (endogenous upregulation) of ChaC1 profoundly sensitized hepatocellular carcinoma (HCC) cells to auranofin (AUR) cytotoxicity. Employing ChaC1-overexpressed models in primary pharmacological screening, we identified AUR as a potent therapeutic candidate under GSH-depleted conditions. This discovery prompted a secondary screening, revealing proteasome inhibitors as robust endogenous ChaC1 inducers. Furthermore, as expected, PI-mediated ChaC1 induction largely enhanced AUR’s anti-cancer activity in HCC cells. Taken together, our ChaC1-based drug screening strategy delineates a synthetic lethal effect between AUR and proteasome inhibitor, proposing a clinically applicable strategy for repurposing these FDA-approved agents in HCC therapy.

Drug repurposing represents one of the hot spots in the research and development of anti-HCC drugs, offering substantial translational advantages through reduced developmental timelines and costs [19, 20]. While the conventional anti-rheumatic agent auranofin (AUR) has demonstrated repurposing potential in HCC treatment [15, 21], and proteasome inhibitors show emerging anti-HCC activity [22, 23], the therapeutic synergism between these clinically established agents remains largely unexplored. Our work addresses this knowledge gap by identifying a synergistic lethality between AUR and proteasome inhibitor in HCC cells. Since both proteasome inhibitors and auranofin have been used in clinic for decades, the direct repurposing potential of these drugs could rapidly translate from bench evidence to bedside application, potentially bringing direct benefit to HCC patients.

Auranofin (AUR) exerts pharmacological activity through targeting cysteine/selenocysteine-dependent antioxidant systems, with thioredoxin reductase 1 (TXNRD1) being its principal therapeutic target [24]. Pharmacodynamic studies reveal a dose-dependent bifurcation in AUR’s efficacy. At submicromolar concentrations, it selectively perturbs redox homeostasis via inhibiting TXNRD1 [25], while high-dose (~5 μM) AUR concurrently impairs proteasomal deubiquitinase (DUB) activity, inducing dual proteotoxic and oxidative stress that drives HCC cell death [17, 26, 27]. Notably, like proteasome inhibitors, high-dose AUR also transcriptionally induces ChaC1, the rate-limiting enzyme in glutathione (GSH) catabolism [27]. It again implicates ChaC1-mediated GSH depletion as a mechanism underlying AUR’s cytotoxic efficacy at elevated concentrations. These observations align with established pharmacological synergism between AUR and GSH biosynthesis inhibitors like buthionine sulfoximine (BSO) in HCC cells [28–30]. Notably, our study provides the first demonstration that ChaC1-catalyzed GSH depletion potentiates AUR-induced lethality, distinct from prior reports of BSO-enhanced cytotoxicity in TXNRD1-deficient/inhibited systems [31, 32]. Altogether, our study introduces enzymatic GSH depletion as a novel strategy in redox-targeted cancer therapy, expanding the therapeutic landscape for AUR-based combination strategies in HCC.

The cell death mode induced by AUR is complex and multifaceted. 20 µM AUR induces ferroptosis through up-regulation of hepcidin expression in HCC cells [33], while 4 ~ 5 µM AUR induces paraptosis in breast cancer cells [27]. Our investigation extends this paradigm by revealing a novel vulnerability in ChaC1-overexpressed HCC systems, where ultralow AUR concentrations (~0.5 μM) elicit non-canonical cell death refractory to pan-caspase inhibitors (apoptosis), necroptosis blockers, or ferroptosis suppressors. Intriguingly, thiol-reducing agents such as NAC and TCEP significantly attenuated AUR-induced cell death, mirroring their inhibitory effects on glucose starvation-induced disulfidptosis in SLC7A11-overexpressed cancer cells [7, 8]. These data implicate disulfide dysregulation as a putative mechanistic nexus between GSH depletion and AUR cytotoxicity, though critical mechanistic distinctions remain to be resolved. Specifically, whether ChaC1/AUR-driven cytotoxicity manifests hallmark disulfidptosis features including thioredoxin oxidation state and disulfide-bonded aggregates requires systematic validation.

In conclusion, this study explores a ChaC1-centric pharmacological discovery platform that identifies proteasome inhibitor and auranofin combination therapy as a mechanistically rational anti-HCC strategy. And we show that PI-mediated ChaC1 induction synergizes with AUR to create a therapeutic vulnerability via dual axes: (1) catastrophic collapse of free thiol homeostasis, and (2) activation of pro-death networks exemplified by DDIT4 upregulation (Fig. 6J). Together, our findings provide both methodological innovation in drug repurposing screening and immediate translational potential for HCC combination regimens.

## Materials and methods

### Cell culture

Huh7, Hep3B and PLC/PRF/5 cells were purchased from Cell Bank of Chinese Academy of Sciences. These cells were cultured at 37°C in an incubator with 5% CO_2_.

### Reagents and antibodies

DiscoveryProbe^TM^ FDA-approved compound library (L1021) was purchased from APEx-BIO (Houston, TX, USA). Auranofin (#HY-B1123), bortezomib (#HY-10227), carfilzomib (#HY-10455), TCEP hydrochloride (#HY-W011500) and N-Acetyl-L-cysteine (#HY-B0215) were purchased from MedchemExpress (Shanghai, China). Ferrostatin-1 (#SML0583-5MG), Z-VAD-FMK (#V116-2MG), chloroquine diphosphate salt (#C6628-25G) and necrostatin-1 (#N9037-10MG), deferoxamine mesylate salt (#D9533-1G) were from Sigma (St. Louis, MO). Cycloheximide (#66-81-9) was purchased from Cell Signaling Technology (Danvers MA). Anti-ATF4 (#10835-1-AP), anti-Nrf2 (#16396-1-AP), anti-DDIT4 (#10638-1-AP) and anti-ChaC1 (#15207-1-AP) antibodies were obtained from Proteintech (Wuhan, China). Anti-ATF3 (#HPA001562) antibody was from Sigma (St. Louis, MO). Anti-β-actin (#HC201) and anti-GAPDH (#HC301) antibodies were purchased from Transgen (Beijing, China).

### In-vitro gene deletion by CRISPR-Cas9 gene editing

ATF4-KO and ATF3-KO Huh7 cells were generated as previously described [34]. Briefly, sgRNAs were identified on the website of Prof. Zhang’s Lab (http://www.genome-engineering.org/crispr). Plasmids expressing Cas9 and sgRNAs were transfected into Huh7 cells. Single-cell clones were picked up, examined by sequencing, and verified by immunoblotting.

### Cell death assay

Cells were stained with 1 µg/mL propidium iodide, and then analyzed using flow cytometry (FACS Canto II, BD Biosciences, Heidelberg, USA).

### Measurement of reactive oxygen species (ROS) generation

The cells were stained with 30 µM DCFH-DA (#HY-D0940) from MedchemExpress (Shanghai, China) and incubated at 37°C for 30 min, washed twice with PBS. Signal was detected by flow cytometry (LSR FortessaX-20, BD Biosciences, USA).

### Measurement of intracellular GSH levels

Total glutathione (T-GSH) levels were measured by Tietze’s recycling assay [35]. Cells were homogenized with the extraction buffer (0.1% Triton X-100 and 0.6% sulfosalicylic acid in potassium phosphate buffer) and centrifuged at 3,000 g and 4°C for 10 min. The supernatant was used to measure the total GSH levels. Alternatively, T-GSH or oxidized GSH (GSSG) were measured by Total Glutathione/Oxidative Glutathione Assay Kit (Nanjing Jiancheng Bioengineering Institute, #A061-1-2) according to the manufacturer’s instruction.

### CCK8 assay

After cells were treated, the cell viability was tested with the TransDetect Cell Counting Kit (#FC101) which was obtained from Transgen (Beijing, China). According to the manufacturer’s instructions, CCK8 solution (10% of the total volume of culture medium) was added into each well. After incubation at 37°C for 1 h, plates were measured on a microplate reader (Thermo Scientific Multiskan GO) at a wavelength of 450 nm. DMEM medium containing 10% FBS was used to remove the background noise.

### Synergy determination with SynergyFinder

The online SynergyFinder software (https://synergyfinder.fimm.fi) was used to calculate drug synergy score with the “inhibition index” (the inhibition index = 100 - Cell Viability) by the response surface model and zero interaction potency (ZIP) calculation method. ZIP synergy scores less than -10 were considered antagonistic, scores from -10 to 10 were considered additive and scores greater than 10 were considered strong synergistic (PMID: 35580060). Heatmaps of drug combination responses were also plotted to assess the therapeutic significance of the combination.

### Western blotting analysis

Cells were homogenized in RIPA buffer containing protease and phosphatase inhibitors. Proteins were separated using SDS-PAGE, transferred to PVDF membranes, and blocked with 5% milk in TBST. The membranes were incubated with a primary antibody solution (1:1000 in TBST with 5% BSA) overnight and with HRP-conjugated secondary antibodies (1:5000) for 1 h. Then, the membranes were reacted with ECL (Millipore) and visualized using Molecular Imager ChemiDoc XRS+ from Bio-Rad.

### Quantitative real-time PCR

RNA was extracted with Trizol (#15596018, Ambion) and first-strand complementary DNA was prepared using the GoScript™ Reverse Transcription System (#A5001, Promega). Then cDNA was amplified with FastStart Universal SYBR Green Master (Rox) (#04913914001, Roche) and detected by Applied Biosystems of Life Technologies. The mRNA levels of the target genes were normalized to those of GAPDH.

### Proteomic analysis

Proteomic study was performed as described [36]. In brief, the peptide samples were resolved in 2% ACN/0.1% FA for LC-MS analysis. The LC used was an EASY-nLC 1200 system (Thermo Fisher Scientific, San Jose, CA) harboring a home-pulled emitter integrative 75 µm by 35 cm C18 column packed with 1.8 µm 120 Å C18 material (Welch, Shanghai, China). The protein sequence files used for DDA database searches excluding decoy sequences were input to DIA-NN (v1.8) [37]. The precursor intensities were input into the Perseus [38] software and log2 transformed.

### RNA interference

To generate gene knockdown cells, Huh7 cells were infected with shRNA-expressing lentiviruses with 4 μg/mL polybrene.

### Primers

The primers used in this study are shown below.

**Table:** 

qRT-PCR primers		
Genes	Forward Primer (5’-3’)	Reverse Primer (5’-3’)
*ChaC1*	TGACGCTCCTTGAAGATCATGAG	CCAGACGCAGCAAGTATTCAAG
*Gapdh*	ATTCCACCCATGGCAAATTC	GATGGGATTTCCATTGATGACA
*Ddit4*	GGTTTGACCGCTCCACGAG	GGTAAGCCGTGTCTTCCTCC
**shRNA sequences (5’-3’)**		
shChaC1	GACGCTCCTTGAAGATCATGAGG	
shDDIT4-#1	TGATGCCTAGCCAGTTGGTAA	
shDDIT4-#2	CCTGAGGATGAACACTTGTGT	
**CRISPR/Cas9 sgRNA sequences (5’-3’)**		
sgATF3	TTTGTGATGGACACCCCGAGGGG	
sgATF4	GGATTTGAAGGAGTTCGACTTGG	

### Statistical analysis

The data are presented as the means ± SEM (standard error of the mean) of three independent experiments. Unpaired Student’s t tests were used to compare the means of two groups. For samples more than two groups, a one-way ANOVA analysis was performed, followed by the recommended post hoc tests using GraphPad Prism software. For two independent variables comparison, a two-way ANOVA test was used. *P* < 0.05 was considered statistically significant. Where applicable, statistical significance is denoted by *: *P* < 0.05; **: *P* < 0.01; ***: *P* < 0.001.

## Supplementary information

Supplementary Figure legends

Supplementary Figure 1

Supplementary Figure 2

Supplementary Figure 3

Supplementary Figure 4

Supplementary Figure 5

Supplementary Figure 6

Supplementary Figure 7

## Data Availability

Original data are available upon request. Uncropped original western blots are shown in the ‘Supplementary Material’.
