# Supplementary material for: ChaC1-based drug screenings identify a synergistic lethal effect of auranofin and proteasome inhibitors in hepatocellular carcinoma cells
Source: Cell Death Discov. 2025 Nov 17;11:532. doi: 10.1038/s41420-025-02838-6 (PMC12624118; doi:10.1038/s41420-025-02838-6)
Supplement: Supplementary file 1 — Supplementary Figure legends [file 41420_2025_2838_MOESM1_ESM.docx]

**Supplementary Figure legends**

**Supplementary Figure 1. The IC50 of AUR was reduced nearly 10-fold in Huh7 cells infected with Ad-ChaC1**

Huh7 cells were infected with Ad-NC or Ad-ChaC1 for 24 hours. And then cells were treated with increasing doses of AUR for 24 hours. Cell viabilities were measured by CCK8 assay.

**Supplementary Figure 2. Co-treatment of AUR with either Ad-ChaC1 or BTZ largely induces DDIT4 expression in Hep3B and PLC/PRF/5 cells**

(A, B) Hep3B (A) and PLC/PRF/5 (B) cells were infected with Ad-NC or Ad-ChaC1 (1×10^8^ pfu/mL) for 12 hours. And then cells were treated with 0.5 µM AUR for 24 hours. After treatment, the expression levels of DDIT4 and ChaC1 were determined by Western Blotting.

**Supplementary Figure 3. Trolox and MitoTempo do not suppress the cell death induced by co-treatment of AUR and Ad-ChaC1**

Huh7 cells were infected with 1×10^8^ pfu/mL Ad-ChaC1 for 24 hours. Cells were treated with or without 5 mM NAC, 2 mM Trolox or 400 µM MitoTempo for 1 hour, and then treated with 0.5 µM AUR for 24 hours. Cell viabilities were measured by CCK8 assay.

**Supplementary Figure 4. Co-treatment of Ad-ChaC1 and AUR dramatically decreases the ratio of GSH/GSSG in HCC cells**

(A, B) Huh7 (A) or PLC/PRF/5 (B) cells were treated with AUR, Ad-NC, Ad-ChaC1, Ad-ChaC1-mut (AUR: 0.5μM; Ad-NC, Ad-ChaC1 and Ad-ChaC1-mut: 1×10^8^ pfu/mL) or their combination. After treatment, cellular total glutathione (T-GSH), reduced glutathione (GSH), oxidized glutathione (GSSG) and GSH/GSSG ratio were measured and calculated by Total Glutathione/Oxidative Glutathione Assay Kits.

**Supplementary Figure 5. ChaC1-induction-based drug screenings find that proteasome inhibitors induce endogenous ChaC1 expression**

(A, B) Huh7 cells were treated with 1 µM drugs from DiscoveryProbe^TM^ FDA-approved compound library of Plate L1021-02 (A) and L0121-03 (B) for 12 hours. The protein expression levels of ChaC1 were determined by western blotting.

**Supplementary Figure 6. Heatmap of BTZ and AUR combination responses**

Cells were treated with either single inhibitors (AUR: 0.5 μM, 1 μM, 2 μM; BTZ: 12.5 nM, 25 nM, 50 nM, 100 nM) or their combinations. After 48 hours treatment, cell viabilities were measured by CCK8 assay. ZIP synergy scores were calculated using Synergyfinder software. Scores > 10 were considered strong synergistic. The gradation of the red regions indicates the intensity of synergism. The white rectangle indicates the concentrations encompassing the region of highest synergy.

**Supplementary Figure 7. Co-treatment of BTZ and AUR dramatically decreases the ratio of GSH/GSSH in HCC cells**

(A, B) Huh7 (A) or Hep3B (B) cells were treated with AUR (1 µM), BTZ (25 nM) or their combination. After 36h treatment, cellular total GSH (T-GSH), GSH, GSSG and GSH/GSSG ratio were measured and calculated by Total Glutathione/Oxidative Glutathione Assay Kits.
