# Supplementary figures and images for: ChaC1-based drug screenings identify a synergistic lethal effect of auranofin and proteasome inhibitors in hepatocellular carcinoma cells
Source: Cell Death Discov. 2025 Nov 17;11:532. doi: 10.1038/s41420-025-02838-6 (PMC12624118; doi:10.1038/s41420-025-02838-6)

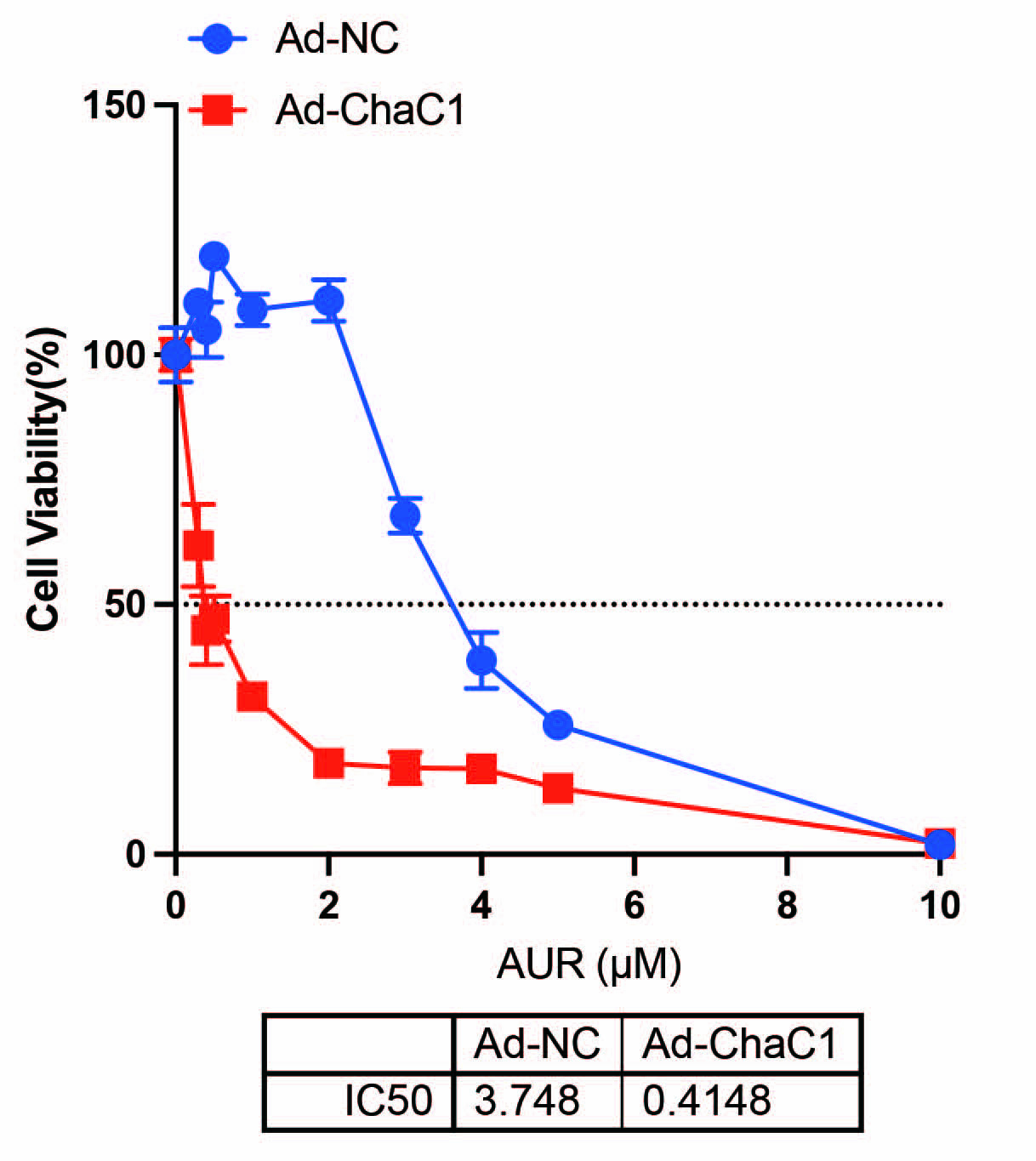

Supplement: Supplementary file 2 — Supplementary Figure 1 [file 41420_2025_2838_MOESM2_ESM.jpg]

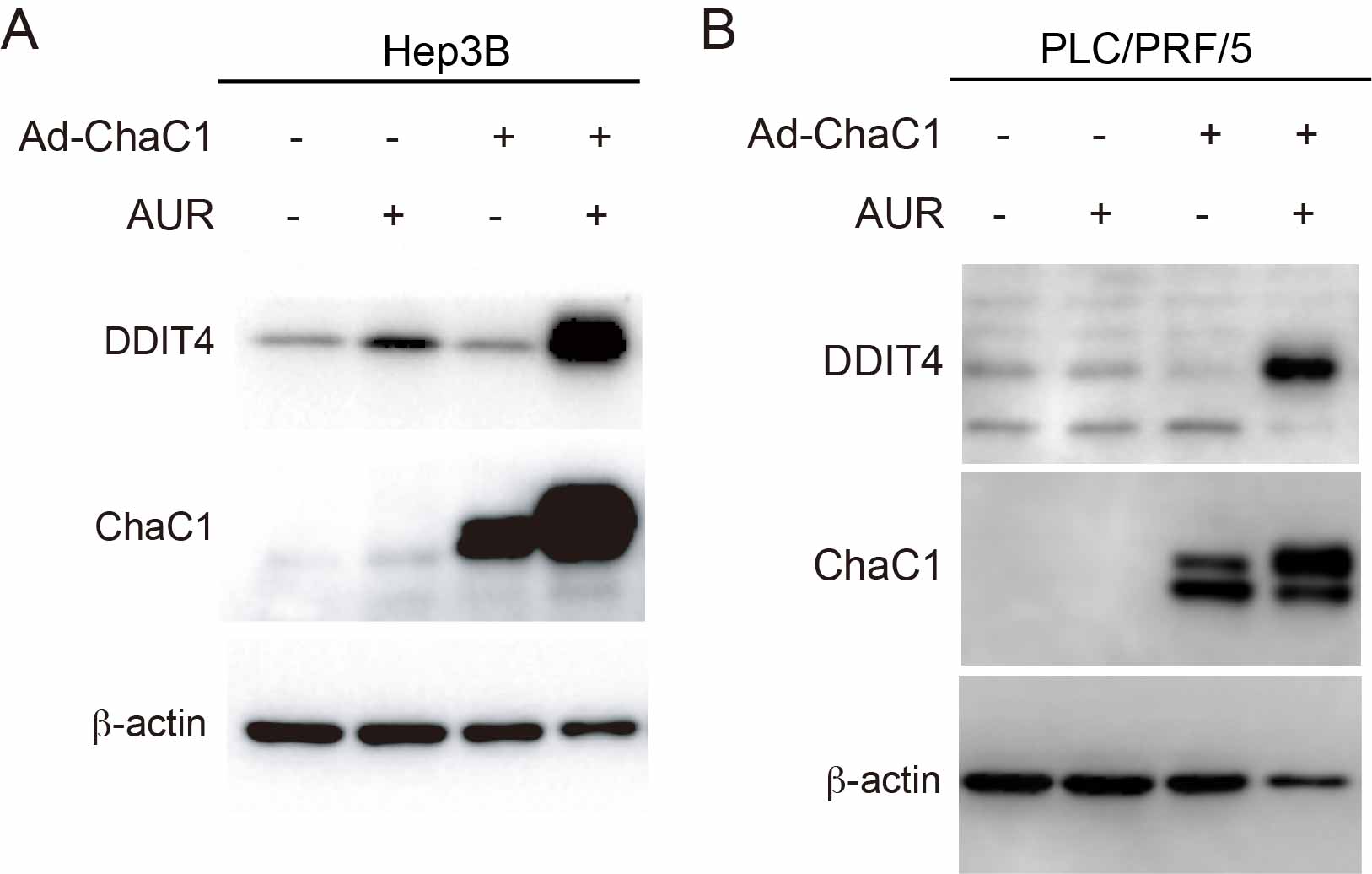

Supplement: Supplementary file 3 — Supplementary Figure 2 [file 41420_2025_2838_MOESM3_ESM.jpg]

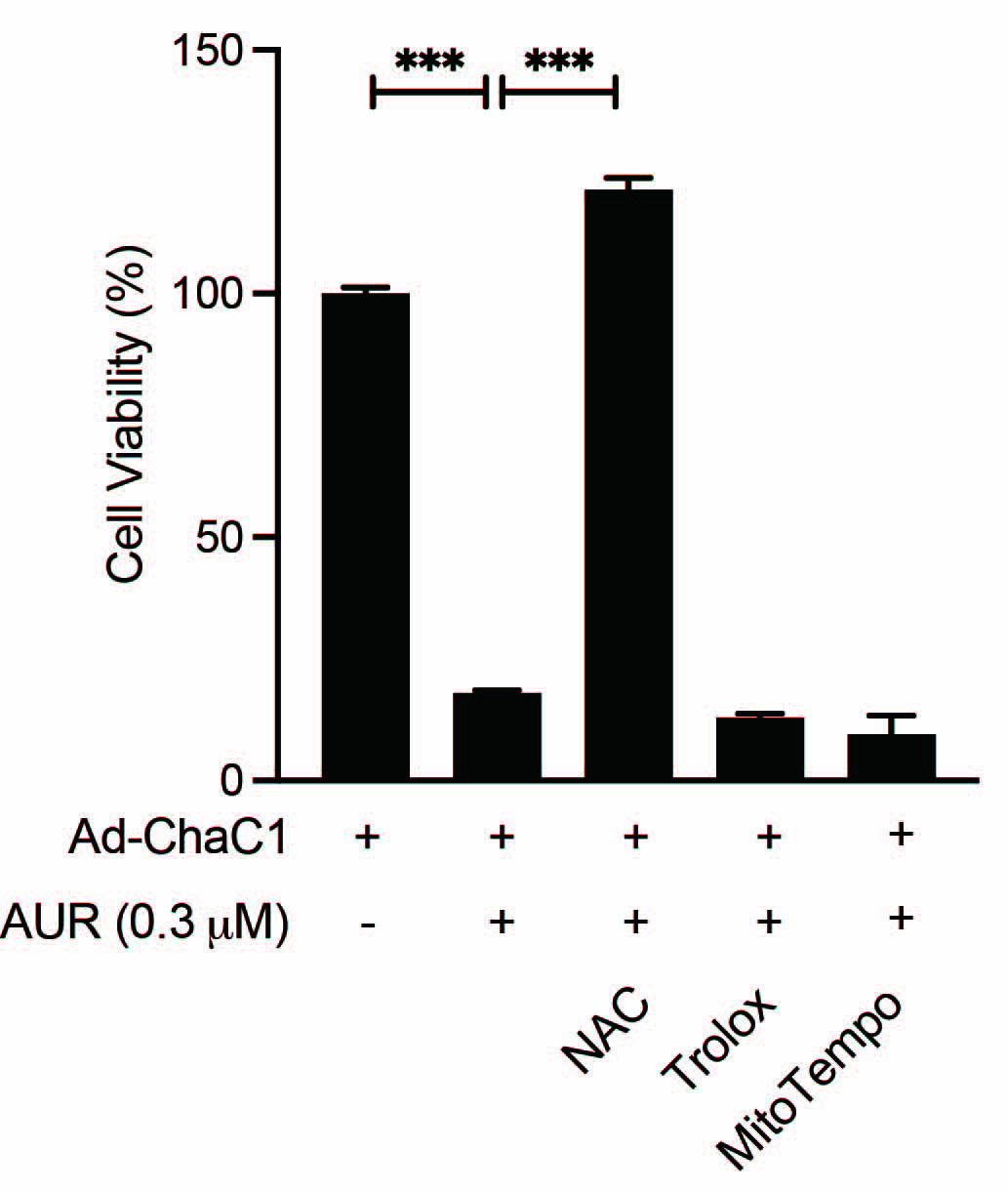

Supplement: Supplementary file 4 — Supplementary Figure 3 [file 41420_2025_2838_MOESM4_ESM.jpg]

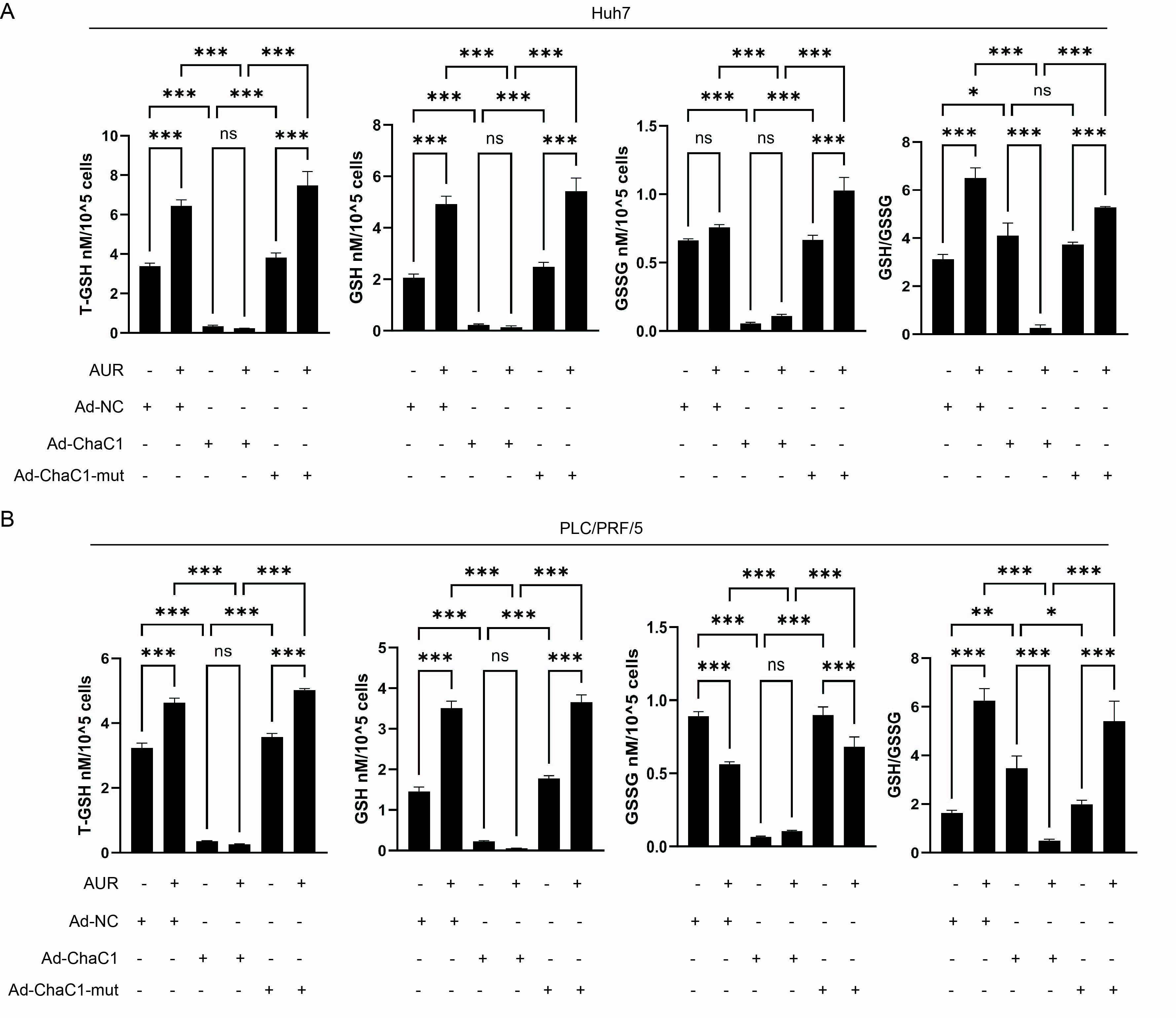

Supplement: Supplementary file 5 — Supplementary Figure 4 [file 41420_2025_2838_MOESM5_ESM.jpg]

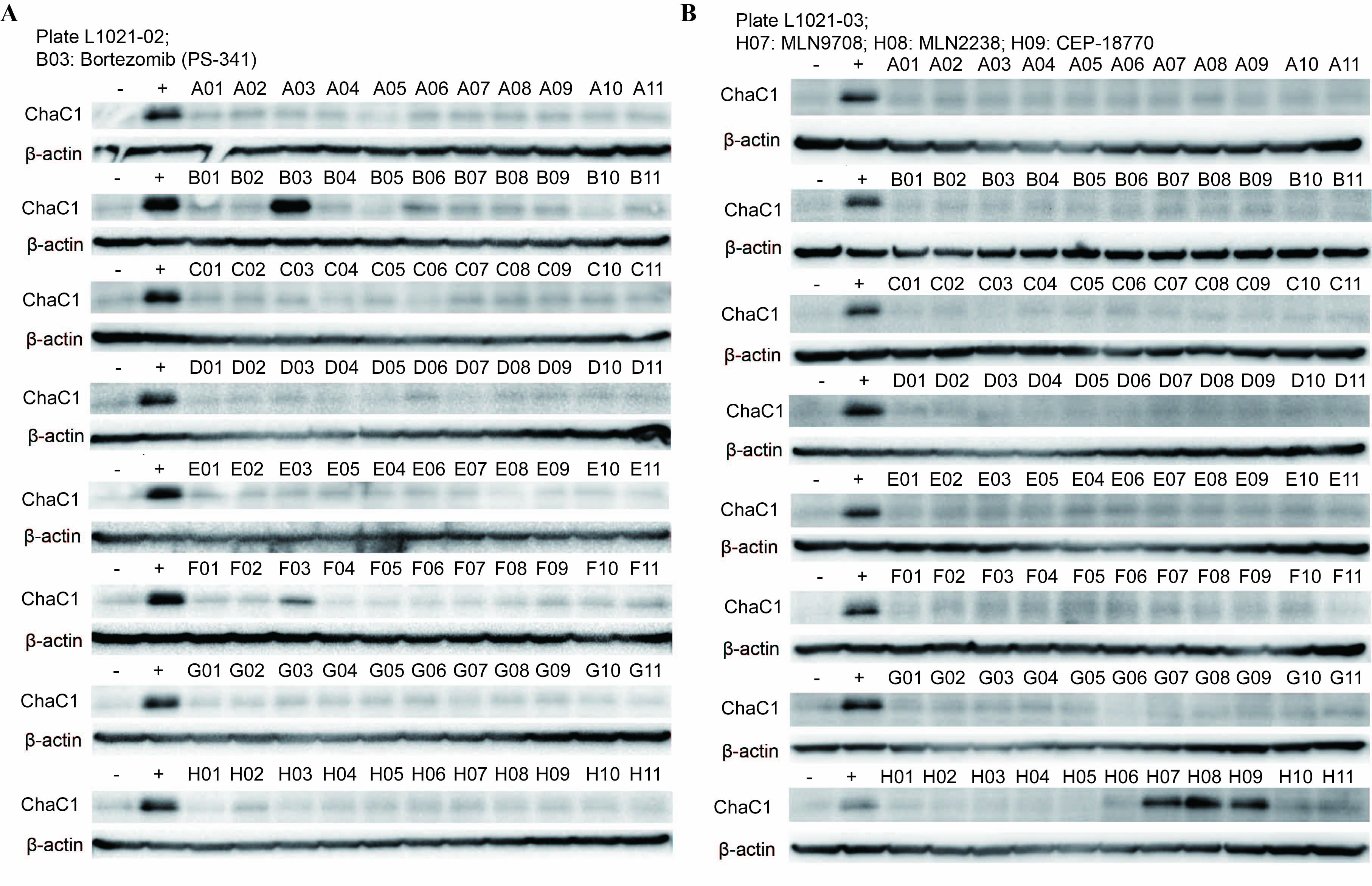

Supplement: Supplementary file 6 — Supplementary Figure 5 [file 41420_2025_2838_MOESM6_ESM.jpg]

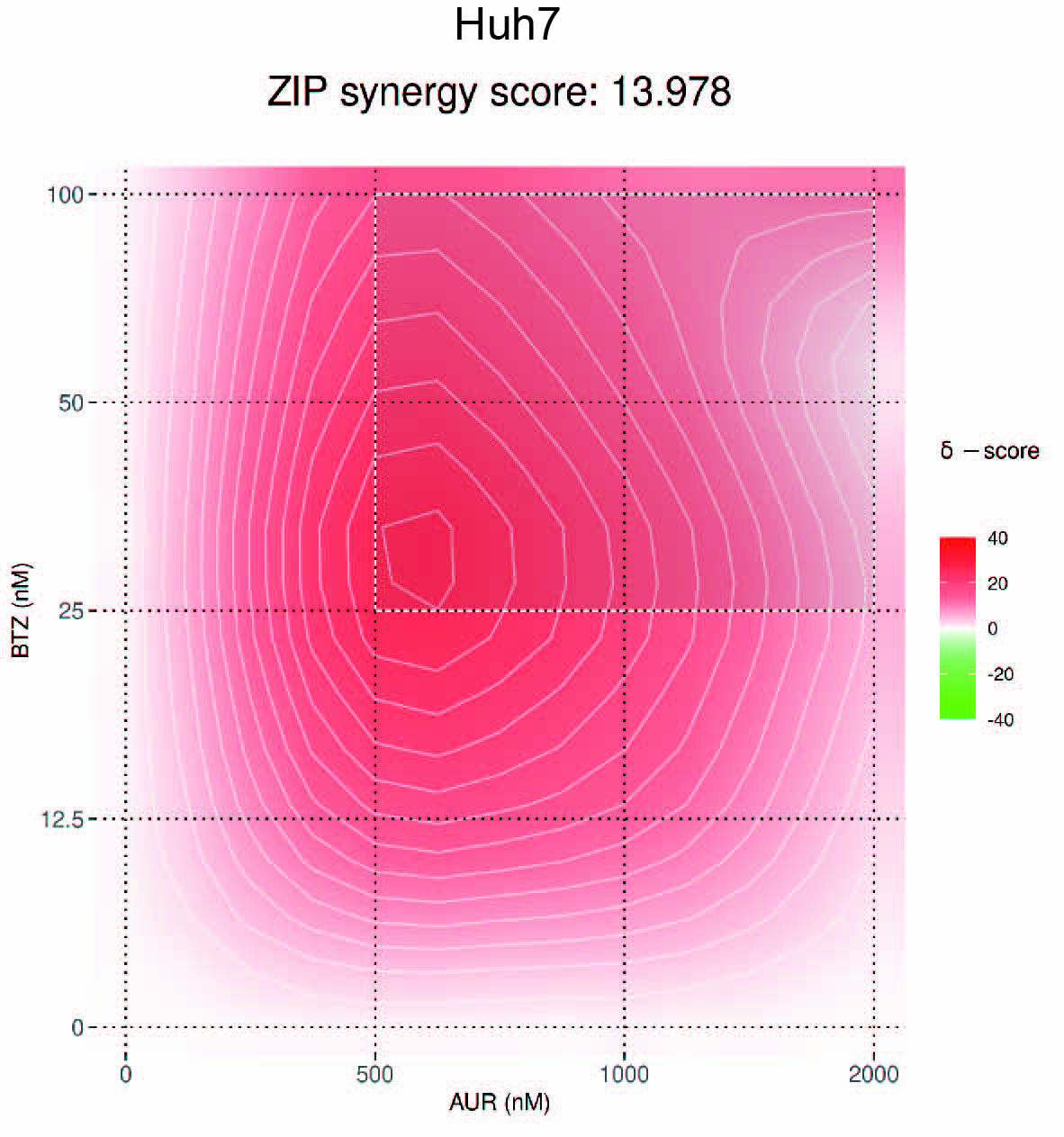

Supplement: Supplementary file 7 — Supplementary Figure 6 [file 41420_2025_2838_MOESM7_ESM.jpg]

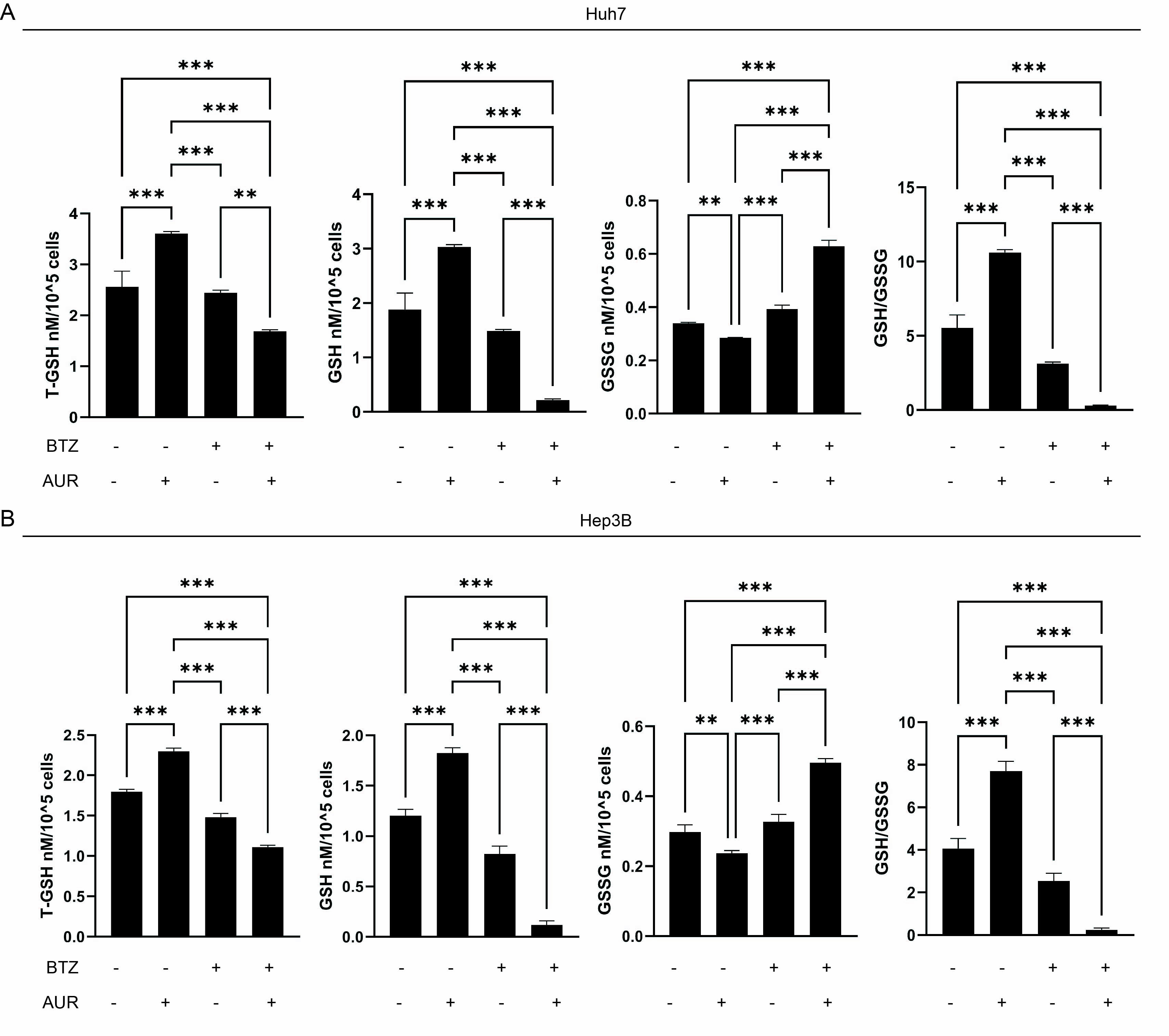

Supplement: Supplementary file 8 — Supplementary Figure 7 [file 41420_2025_2838_MOESM8_ESM.jpg]
